# Supplementary material for: Liquid Biopsies Poorly miRror Renal Ischemia-Reperfusion Injury
Source: Noncoding RNA. 2023 Apr 1;9(2):24. doi: 10.3390/ncrna9020024 (PMC10141369; doi:10.3390/ncrna9020024)
Supplement: Supplementary file 1 [file ncrna-09-00024-s001.zip › Table S1.pdf]

Table S1. Basic rat phenotypes collected 24 hours after reperfusion (or corresponding sham surgery). *Urine volumes represent the volume of urine collected from metabolic caging in the period between recovery from surgical anesthesia and endpoint at 24 hours post-reperfusion (or sham surgery)

Rat ID	Study Group	Body Weight (g)	Right Kidney Weight (g)	Left Kidney Weight (g)	Urine Volume (mL)
IRRCS_1	IR	356.0	1.936	1.759	7
IRRCS_2	IR	301.2	1.157	1.2061	4.5
IRRCS_3	IR	299.6	1.246	1.2403	10
IRRCS_7	IR	310.5	1.2028	1.2750	4
IRRCS_10	IR	325.4	1.5221	1.3541	7.5
IRRCS_11	IR	326.3	1.2108	1.5696	4
IRRCS_12	IR	329.6	1.3621	1.3205	9
IRRCS_4	Sham	322.8	1.975	1.0997	5
IRRCS_5	Sham	292.9	0.9165	0.893	4
IRRCS_6	Sham	303.0	1.0353	1.0258	5
IRRCS_8	Sham	304.2	1.229	1.2045	3.5
IRRCS_9	Sham	298.3	1.0418	1.0433	4
