# Supplementary material for: Liquid Biopsies Poorly miRror Renal Ischemia-Reperfusion Injury
Source: Noncoding RNA. 2023 Apr 1;9(2):24. doi: 10.3390/ncrna9020024 (PMC10141369; doi:10.3390/ncrna9020024)
Supplement: Supplementary file 1 [file ncrna-09-00024-s001.zip › Table S3.pdf]

Table S3. DE miRNAs from IR vs. sham comparisons with 100% seed sequence homology between rat (rno), mouse (mmu) and human (hsa). Change (∆) in miR abundance with IR indicated by arrows with (↓ = decreased and ↑ = increased).

Sample Type	DE miRs	∆ with IR (vs. Sham)	log2FC
Plasma	hsa-miR-103b	↑	2.8621
	rno-miR-10a-3p	↑	2.2347
	rno-miR-10a-5p	↑	1.3384
	rno-miR-130a-3p	↑	1.8197
	rno-miR-151-5p	↑	0.8299
	rno-miR-200a-5p	↑	1.9999
	rno-miR-218a-1-3p	↑	2.7310
	rno-miR-219a-2-3p	↑	4.0006
	rno-miR-22-5p	↑	1.4025
	rno-miR-3085	↑	2.8706
	rno-miR-320-3p	↑	1.3973
	rno-miR-760-3p	↑	1.5145
	rno-miR-99b-5p	↑	1.1626
	rno-miR-365-5p	↑	2.2419
	rno-miR-219b	↑	3.8698
	rno-miR-3556a	↑	1.9021
	rno-miR-203b-5p	↑	2.9047
	rno-miR-429	↑	1.8244
	rno-miR-203a-5p	↑	3.6243
	rno-miR-203b-3p	↑	3.6009
	rno-miR-192-5p	↑	2.3489
	rno-miR-378b	↑	1.4002
	rno-miR-504	↑	2.7498
	rno-miR-183-3p	↑	3.7737
	rno-miR-125a-5p	↓	-1.1335
	rno-miR-142-3p	↓	-1.4314
	rno-miR-143-3p	↓	-1.2803
	rno-miR-145-5p	↓	-0.7914
	rno-miR-150-5p	↓	-1.1176
	rno-miR-199a-3p	↓	-1.2610
	rno-miR-19a-3p	↓	-1.9437
	rno-miR-19b-3p	↓	-1.7820
	rno-miR-335	↓	-1.4600
	rno-miR-340-5p	↓	-1.5643
	rno-miR-455-5p	↓	-1.9201
	rno-miR-497-5p	↓	-1.2544
	rno-miR-133a-3p	↓	-1.4758
	rno-miR-133a-5p	↓	-1.4729
	rno-miR-101a-5p	↓	-1.6440
	rno-miR-362-3p	↓	-1.6050
	rno-miR-488-3p	↓	-1.6699
	rno-miR-3559-5p	↓	-1.5638
	rno-miR-133c	↓	-1.2915

Sample Type	DE miRs	∆ with IR (vs. Sham)	log2FC
Urine	rno-miR-22-3p	↑	0.6700
	rno-miR-877	↑	2.4805
	let-7e-5p	↓	-1.4761
	miR-196b-5p	↓	-1.4190
	miR-23b-3p	↓	-2.4490
	miR-26a-5p	↓	-1.3099
	miR-27b-3p	↓	-1.0790
	miR-351-5p	↓	-2.7794
Cortex	let-7a-5p	↑	0.3956
	let-7c-5p	↑	0.7022
	rno-miR-132-3p	↑	2.2297
	rno-miR-146b-5p	↑	2.1612
	rno-miR-147	↑	1.3324
	rno-miR-155-5p	↑	1.4300
	rno-miR-18a-5p	↑	1.8245
	rno-miR-20a-5p	↑	0.8320
	rno-miR-212-3p	↑	1.9420
	rno-miR-21-3p	↑	1.6191
	rno-miR-21-5p	↑	1.3052
	rno-miR-221-3p	↑	0.8981
	rno-miR-221-5p	↑	0.7743
	rno-miR-222-3p	↑	1.0252
	rno-miR-27a-5p	↑	1.3213
	rno-miR-3473	↑	2.3535
	rno-miR-34c-5p	↑	1.2235
	rno-miR-3596a	↑	0.3480
	rno-miR-449a-5p	↑	1.4869
	rno-miR-672-5p	↑	0.6846
	rno-miR-708-5p	↑	1.0462
	rno-miR-92a-1-5p	↑	1.3291
	rno-miR-98-5p	↑	0.5486
	hsa-miR-486-5p	↓	-0.9168
	rno-miR-125-b-2-3p	↓	-0.6346
	rno-miR-144-5p	↓	-0.9183
	rno-miR-194-5p	↓	-0.6254
	rno-miR-200-3p	↓	-0.4505
	rno-miR-201-5p	↓	-0.5956
	rno-miR-30c-5p	↓	-0.7125
	rno-miR-30e-5p	↓	-0.5930
	rno-miR-3585-5p	↓	-0.8564
	rno-miR-499-5p	↓	-0.7358
