# Supplementary material for: Liquid Biopsies Poorly miRror Renal Ischemia-Reperfusion Injury
Source: Noncoding RNA. 2023 Apr 1;9(2):24. doi: 10.3390/ncrna9020024 (PMC10141369; doi:10.3390/ncrna9020024)
Supplement: Supplementary file 1 [file ncrna-09-00024-s001.zip › Table S4.pdf]

**Table S4.** DE miRNAs from IR vs. sham comparisons common between plasma and urine samples. Decrease in miR abundance with IR indicated in green and increase in miR abundance with IR within indicated in red. As in Table S1, these miRs share 100% seed sequence homology between rat (rno), mouse (mmu) and human (hsa).

| Overlapping miRs | Plasma (log2FC) | Urine (log2FC) |
|---|---|---|
| rno-miR-378a-3p | 1.4357 | 0.8812 |
| rno-miR-423-3p | 0.9458 | 1.3515 |
| rno-miR-3556b | 1.7866 | 1.6383 |
| rno-miR-3557-5p | 1.3752 | 0.9581 |
| rno-miR-3590-5p | 2.1782 | 1.8741 |
| rno-miR-215 | -1.1731 | 3.0324 |
