# Supplementary material for: Liquid Biopsies Poorly miRror Renal Ischemia-Reperfusion Injury
Source: Noncoding RNA. 2023 Apr 1;9(2):24. doi: 10.3390/ncrna9020024 (PMC10141369; doi:10.3390/ncrna9020024)
Supplement: Supplementary file 1 [file ncrna-09-00024-s001.zip › Table S5.pdf]

Table S5. DE miRNAs from IR vs. sham comparisons common between plasma and cortex samples. Within sample type, a decrease in miR abundance with IR indicated in green and increase in miR abundance with IR within indicated in red. As in Table S1, these miRs share 100% seed sequence homology between rat (rno), mouse (mmu) and human (hsa).

Overlapping miRs	Plasma (log2FC)	Cortex (log2FC)
rno-miR-466b-2-3p	1.5071	1.547
rno-miR-144-3p	-1.7298	-1.2127
rno-miR-3553	4.4954	-1.2531
rno-miR-182	4.6282	-1.2651
rno-miR-183-5p	4.5408	-1.3014
rno-miR-192-5p	2.3489	-0.6567
rno-miR-203a-3p	2.3999	-0.6541
rno-miR-375-3p	1.9447	-1.0184
